# A Novel Spread Spectrum and Clustering Mixed Approach with Network Coding for Enhanced Narrowband IoT (NB-IoT) Scalability

**DOI:** 10.3390/s20185219

**Published:** 2020-09-13

**Authors:** Emmanuel Migabo, Karim Djouani, Anish Kurien

**Affiliations:** 1 Department of Electrical Engineering, French South African Institute of Technology (F’SATI), Tshwane University of Technology, Pretoria 0001, South Africa; DjouaniK@tut.ac.za (K.D.); KurienAM@tut.ac.za (A.K.); 2 Laboratoire Images, Signaux et Systemes Intelligents (LISSI), University of Paris Est Creteil (UPEC), F-94400 Vitry, France

**Keywords:** spread spectrum, adaptive, frequency hopping, clustering, network scalability, energy efficiency, network reliability, network coding, network lifetime

## Abstract

The Narrowband Internet of Things (NB-IoT) is a very promising licensed Internet of things (IoT) technology for accommodating massive device connections in 5G and beyond. To enable network scalability, this study proposes a two-layers novel mixed approach that aims not only to create an efficient spectrum sharing among the many NB-IoT devices but also provides an energy-efficient network. On one layer, the approach uses an Adaptive Frequency Hopping Spread Spectrum (AFHSS) technique that uses a lightweight and secure pseudo-random sequence to exploit the channel diversity, to mitigate inter-link and cross-technology interference. On the second layer, the approach consists of a clustering and network coding (data aggregation) approach based on an energy-signal strength mixed gradient. The second layer contributes to offload the BS, allows for energy-efficient network scalability, helps balance the energy consumption of the network, and enhances the overall network lifetime. The proposed mixed strategy algorithm is modelled and simulated using the Matrix Laboratory (MATLAB) Long Term Evolution (LTE) toolbox. The obtained results reveal that the proposed mixed approach enhances network scalability while improving energy efficiency, transmission reliability, and network lifetime when compared to the existing spread spectrum only, nodes clustering only, and mixed approach with no network coding approaches.

## 1. Introduction

The Internet of Things (IoT) has changed the way we view and use technology. As consumers, we have come to expect connectivity and access to information wherever we go. According to Information Handling Services (IHS) technology forecast, the Internet of Things (IoT) market is expected to grow to billions of devices by 2020 [1]. Massive connections are expected to respond to different IoT use cases such as smart cities, smart wearables, smart homes, etc. [2]. For these applications, latency-insensitive devices can be positioned in hard-to-reach areas and do not require high throughput or frequent reporting. Therefore, to cope with such tremendous IoT trends, the Third-Generation Partnership Project (3GPP) introduced the Narrowband Internet of Things (NB-IoT) standard as a pioneer communication technology enabler within the licensed band. However, it is still acknowledged that in its current shape and form, the NB-IoT is faced with multiple challenges and design issues, some of which the present research work proposes to address, including the spectrum resource limitation as this remains a key aspect in enabling massive IoT. It is not only important to ensure efficient spectrum sharing to enable network scalability, but at the same time to ensure energy efficiency.

The NB-IoT is categorized as one of the licensed Low-Power Wide-Area Networks (LPWAN) cellular technologies based on cellular Technologies such as the Long-Term Evolution (LTE) and the 5th generation technology (5G) with long-range and low cost. In the LPWAN category, exists other licensed technologies, i.e., Long-Term Evolution Category M1 (LTE-M), and unlicensed technologies, i.e., Long Range (LoRa), SigFox, Ingenu, etc. [3,4,5,6,7], but they are not the focus of the current work since they are not based on cellular technology. The term Narrowband refers to NB-IoT’s bandwidth of maximum 200 kHz thanks to which it can coexist either in the Global System for Mobile Communications (GSM) spectrum or by occupying one of the legacy LTE Physical Resource Blocks (PRBs) as in-band or as guard-band. Since it coexists in the LTE spectrum, NB-IoT follows the legacy LTE numerologies as it uses Orthogonal Frequency Division Multiplexing (OFDM) and Single-Carrier Frequency Division Multiple Access (SC-FDMA) in the downlink and uplink transmission schemes, respectively. Some modifications in the physical (PHY) and medium access control (MAC) layers are implemented to support the long-range massive machine-type (mMTC) connections with low power, low data rates, low complexity, and hence low cost. However, despite its low complexity, this new radio access technology (RAT) delivers better performance in terms of the supported number of devices, and coverage enhancements for latency-insensitive applications with maximum coupling loss (MCL) of about 20 dB higher than LTE (i.e., 164 dB) [3,4,5,8,9,10,11].

The NB-IoT is one of the most prominent IoT technology within the licensed band. Despite all the challenges faced by NB-IoT such as energy efficiency because NB-IoT nodes run on batteries, coverage range, reliability of Uplink (UL) and Downlink (DL) communications, etc, there is still more and more devices being added to the network as the range of applications and needs keeps increasing. This poses a serious challenge when it comes to the NB-IoT network design considerations in terms of its different layers. This work focuses on proposing a novel spread spectrum and clustering combination approach as a mechanism to enhance network scalability of the NB-IoT. The challenge, however, remains to ensure that despite the use of a new mixed approach, the NB-IoT network still maintains an acceptable level of energy efficiency, network reliability, and data rates. Several clustering techniques for wireless IoT exist, however to the best of our knowledge, none simultaneously considers signal strength and remaining energy of nodes as a combined parameter for cluster head selection.

The Frequency Hopping Spread Spectrum remains one of the most reliable and ruggedized options for modern Internet of Things communications. Therefore, despite the rapidly changing technology landscape, the FHSS remains a good choice for long-range communication and has the potential to accommodate the growing number of devices in the field thanks to its ability to efficiently and dynamically share network spectral capacity among devices. This work, therefore, proposes a new FHSS-based spread spectrum approach, adapted to the NB-IoT PHY layer characteristics, which, when coupled with a novel clustering approach, aims at enhancing the NB-IoT network scalability while preserving its energy efficiency and network reliability.

Due to the limited available spectrum (roughly 200 kHz), as allocated to the NB-IoT within its three deployment modes (in-band, standalone, and guard band); one of the key issues is how to efficiently use these resources to support massive IoT devices. Because the NB-IoT technology is deemed to have a higher level of computational complexity at Base Station as compared to its unlicensed band competitors such as the LoRa and Sigfox; the concepts of time offset as well as transmission repetition have been proposed by previous work such as [12,13]. Finally, there is also the fact that frequency band allocation within the cellular network’s band (in-band deployment) or as standalone does often causes non-synchronization among different cells which results in inter-cell interference from the neighbor cells’ LTE users or NB-IoT users.

The above three reasons coupled with the fact that most NB-IoT technology designs, as currently proposed, mainly use simple ALOHA or CSMA based MAC protocols which do not scale well with a large number of connected devices; have been considered by the present study as a trigger for a need for a novel spread spectrum and clustering approach for the NB-IoT. Therefore, the present work proposes a mixed-strategy of frequency hopping spread spectrum and clustering approach to mitigate the inter-cell interference and reduce computational complexity at the BS. This is expected to enhance NB-IoT system designs as a way to enable network scalability, data rate enhancement, and also maintain energy efficiency and network reliability.

The rest of this paper is organized as follows. Section 2 presents a summary of existing spectrum sharing and clustering techniques for NB-IoT as found in the literature. Section 3 presents a detailed development of the proposed mixed spread spectrum and clustering algorithm. Section 4 presents the performance evaluation of the proposed followed by the analysis and discussion of the obtained results. Section 5 concludes the paper and presents future work guidelines.

## 2. Related Work

The sharing of network resources remains a very crucial aspect of NB-IoT network designs. It is a key feature for enhancing network scalability and enabling the connection of massive NB-IoT devices to the network. Various approaches have been previously proposed among which tone allocations [9], Physical Resources Blocks (PRBs) allocation techniques [14], repetition number techniques [12], power configurations and control techniques [15], subframes, or time slots management techniques at Medium Access Layer (MAC) level [16], etc. Therefore, several research works, as discussed in this section, have a special focus on achieving better radio resource management to ensure energy efficiency, high throughput, high spectral capacity, and better coverage. Of these various approaches, this literature review study focuses mainly on two aspects, namely efficient spectrum management and clustering techniques.

### 2.1. Spectrum Sharing Techniques in Licensed Band Iot Systems

**Figure 1:**
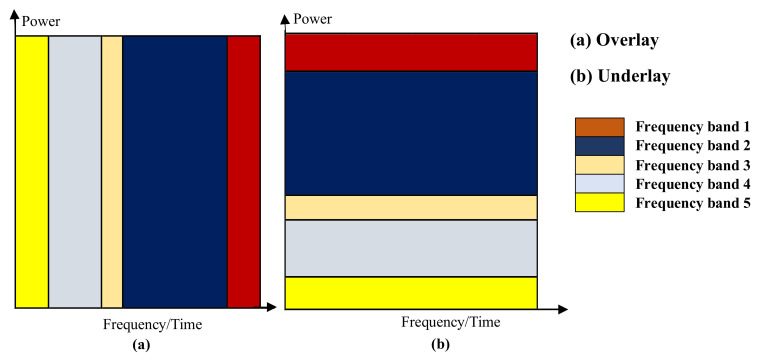
Overlay versus underlay spectrum sharing solutions.

Spectrum sharing is a preferable solution for IoT due to the scarcity of available spectrum resources. In a very special manner, mobile operators are inclined to exploit the existing standards and infrastructures of current cellular networks and deploy licensed IoT such as the NB-IoT within the already congested licensed cellular spectrum [17]. The reason why our study focuses mainly on the NB-IoT, among other licensed IoT systems, is simply that unlike the other licensed systems such as the eMTC which mainly exploits the LTE spectrum, the NB-IoT standard makes previsions not only to exploit the LTE spectrum but all other re-farmed cellular spectra. This leaves enough maneuver for NB-IoT systems designs as compared to other licensed IoT systems. This uniqueness of the NB-IoT spectrum creates room for more efficient spectrum exploitation which in turn translates in the ability to have more connected NB-IoT nodes and better channel capacity utilization. However, this unique benefit requires the use of a well-designed spectrum-sharing algorithm capable of properly mitigating and managing interference while efficiently utilizing the already limited network resources, such as energy. One of the main existing approaches for spectrum sharing in the NB-IoT system designs is the orthogonal spectrum allocation technique. This technique consists of centralizing the management of spectrum management. The advantage of this approach is that it allows cellular network operators to avoid cross-technology interference between NB-IoT network with the current cellular networks (5G, LTE, etc.) on one hand, and on the other hand, the other licensed IoT networks deployed on the same cellular network such as the LTE-Machine Type Communication (LTE-M) that includes the enhance Machine Type Communication network (eMTC). This orthogonal approach is also referred to as overlay spectrum sharing as opposed to the underlay (non-orthogonal Multiple Access (NOMA)) spectrum sharing [18]. In the overlay approach, different users (Cellular User Equipments (UEs), as well as IoT (NB-IoT and LTE-M) nodes, are allocated separate time or frequency resource blocks (RBs) with the same power level, while in the underlay approaches, different users are allocated different power levels but are serviced using the same time or frequency resource blocks [19]. The difference between the two approaches is illustrated in Figure 1.

This study considers the use of a spread spectrum approach as part of the orthogonal frequency sharing techniques because of the nature of the NB-IoT spectrum which does not only comprise of the spectrum as provided by the cellular network to which it is attached but also comprises of other re-farmed cellular spectra. This characteristic provides an opportunity to mitigate interference and therefore, allows us to connect more NB-IoT nodes within a cell. This is possible provided that a smart and well-designed frequency selection algorithm is used. This is one of the objectives pursued by the present study.

Other spectrum sharing techniques used for NB-IoT systems include spectrum sharing techniques similar to those in cognitive radio networks. In most of these cognitive approaches, the licensed IoT networks are considered as secondary cognitive transmitters while the primary transmitters are the UEs of the cellular network to which the IoT network is attached [20,21]. In the cognitive approaches, the NB-IoT nodes (secondary transmitters) may access the primary spectrum if the primary transmitters are not active or are not susceptible to cause significant interference [22]. Most of these cognitive algorithms are designed in such as way that the secondary transmitters (NB-IoT nodes in this case) are regulated by sensing the activities of primary transmissions and at the same time, impose stringent secondary access constraints on some well-defined network performance objectives such as the probability of collision [22,23]. One of the approaches used to minimize secondary interference is the use of Multiple Inputs Multiple Output (MIMO) strategies [24]. However, this approach is not very feasible in the most context of NB-IoT nodes designs because of their limited energy, computational and memory resources. Other approaches even go further by using a dynamical game approach to study the spectrum sharing among a primary user and multiple secondary users [25]. Unfortunately, unlike cognitive radios, in the NB-IoT networks, the primary transmitters are normally the NB-IoT nodes and BSs. Besides, although the cognitive radio like techniques may work for few connected NB-IoT nodes (10 to 20 nodes per cell), they become completely non-feasible for massive NB-IoT systems as the probability of interference rises significantly to the extent of almost nullifying their benefit. Therefore, how Device-to-Device (D2D) networks such as the NB-IoT systems should access the spectrum remains an open problem with its specific challenges as discussed in Section 2.2.

### 2.2. Spread Spectrum Challenges in NB-IoT Systems

It is expected that the ever-expanding number of connected NB-IoT devices will generate a massive volume of data, which has diverse requirements including network lifetime (≥10 years [26]), latency (<4 ms [27]), reliability (SNR ≥−12.6 dB [28]), and throughput(up to 26 kbps in downlink and 66 kbps in uplink [29]). Some of these devices will be dedicated to real-time IoT applications such as remote surgery etc. These specific devices will, therefore, have very low latency requirements which for a given available spectral efficiency accessible to them at a given instant of time, will require a larger bandwidth. These types of applications are the most likely to lose connectivity in a cell as more and more devices join the network. Despite this challenge, it is important to emphasize that the NB-IoT remains the choice of preference for enhanced network scalability [30]. This is because, unlike the unlicensed IoT technologies (Long Range (LoRa), SigFox etc.), it has the benefit of freely leveraging the cellular spread spectrum technology. When considered against other IoT technologies such as the LTE-M technologies, the NB-IoT is more flexible since it can operate in 2G, 3G, 4G, and 5G bands. This eliminates the need for a gateway, which saves costs in the long run. The competitive advantage of NB-IoT technology over the other IoT technologies in terms of achieving network scalability has been emphasized in the previous paragraph. However, it is important to note that the use of the spread spectrum sharing approach for NB-IoT systems has challenges of its own. A key issue with the use of spread spectrum techniques is how they afford protection against interfering signals with finite (limited) power [31]. As more and more NB-IoT devices and cellular UEs attempt to simultaneously access the uplink NB-IoT channel, the likelihood of their power as distributed across the channel to overlap increases and eventually results in interference. Hence, one key design aspect when it comes to the spread spectrum algorithm lies in the random sequence generation approach. The challenge for the random sequence generation approach is to simultaneously achieve repeatability, to pass all statistical tests of randomness, to achieve insensitivity to the seed values and finally to ensure that the finite period after which the sequence repeats itself is much longer than the amount of random number needed at a given time as the network grows (as the number of connected NB-IoT devices increases). Therefore, the present paper proposes an Adaptive Frequency Hoping Spread Spectrum technique capable dynamically adjusting the random sequence number taking into account the amount of remaining energy of the NB-IoT nodes in the network.

### 2.3. Clustering Approaches for Energy-Efficient NB-IoT

Clustering techniques have proven to be beneficial in terms of enhancing the energy efficiency of the Internet of Things networks. They have also proven to be very effective in balancing the energy consumption of the network. This is because they allow an offload of the Bases Station which, despite the increasing number of nodes, results in more reliable communications. However, one of the major challenges of clustering has been the fact that the use of fixed cluster heads has proven to shorten the life cycle of the network. This is because most NB-IoT nodes are energy limited as they run on batteries, Therefore, using some selected NB-IoT nodes as relays to a group of others poses a serious energy burden to these which eventually die. As the later die, this results in the inability of some NB-IoT nodes to reach the BS. This problem, also often qualified as a “hotspot problem” has triggered various research interests within the community of scientists. The present section takes us through the different clustering approaches as applied to the specific case of NB-IoT systems in an attempt to resolve this issue.

Several energy-efficient techniques have been developed for IoT networks in general and NB-IoT networks in particular. Some of the key techniques include but are not limited to the following,

- Low-Energy Adaptive Clustering Hierarchy (LEACH) and its variants: LEACH is s a hierarchical technique that consists of nodes transmitting data packets to cluster heads which first aggregate the received packets, compress the aggregated data before forwarding it to the base station (sink). In LEACH, every single node uses a stochastic algorithm at each transmission round to determine if it is its turn to become a cluster head. Nodes that have been cluster heads cannot become cluster heads again for *P* rounds, where *P* is the desired percentage of cluster heads. Thereafter, each node has a 1/P probability of becoming a cluster head again. At the end of each round, each node that is not a cluster head selects the closest cluster head and joins that cluster. The cluster head then creates a schedule for each node in its cluster to transmit its data. One major weakness of LEACH as applied to NB-IoT systems is that it is only reliable under the condition that the BS is within the radiation range of each NB-IoT node. Due to their geographical dispersion and mostly randomized locations in some applications, some NB-IoT nodes happen to be at non-communication range to the BS when their radio is operated in low-power (energy saving) modes. Therefore, most of the time, if the nodes’ firmware is smart enough, it adjusts the radio transmission power to its maximum for the communication round in which a specific node plays the role of cluster head. Considering the multiple numbers of data packets from all the neighboring nodes and assuming that more and more NB-IoT nodes are added to the network (massive NB-IoT systems), that specific NB-IoT node depletes its residual energy and dies, this despite any form of network coding technique applied to save energy. There are other variants of the LEACH protocol that are proposed as a mechanism to enhance its performance. These include the LEACH centralized (LEACH-C) protocol [32]. The LEACH-C scheme is meant to improve the CH allocation in LEACH by using a centralized control algorithm to distribute the CH nodes throughout the system. In LEACH-C, the BS computes the average residual energy of the nodes across the network and only allows the nodes with residual battery powers equivalent to the computed average residual energy to work as CH in each round. The LEACH with fixed clusters (LEACH-F) is another centralized variant of LEACH.18 In LEACH-F, the setup phase is not required at all rounds, only the CH position rotates among the nodes. The data fusion oriented clustered routing protocol based on LEACH (DF-LEACH) is another variant of LEACH protocol [33]. DF-LEACH aims to facilitate both global energy efficiency and reduced energy dissipation at the CHs [34]. In this scheme, data fusion is performed in a hop-by-hop mode among the CHs, before transmission to the BS.
- The Energy-Efficient Hierarchical Clustering (EEHC): This clustering approach as proposed by [35] uses Voronoi cells approach to classify (group) nodes into clusters. The cluster head selection is based on residual energy. The nodes with the most residual energy within each cluster is selected as the cluster head. The selected cluster head is then used to allocate time slots to the other nodes within the cell based on the hidden Markov prediction model. The benefit of the approach is that it has proven to enhance the overall energy efficiency of the NB-IoT network. However, since it does not consider the signal strength parameter in the selection of the cluster heads, some packets do not always get delivered to the BS. This network reliability reduction results in re-transmission attempts which happens to be energy expensive and counter-effects the impact of clustering on the overall energy efficiency of the network.
- Energy-Efficient Unequal Clustering (EEUC): Authors in [36] propose an algorithm that minimizes the number of CHs with full coverage and connectivity. This extra optimization performed on the number of selected CHs is aimed at reducing the energetic cost of communication in the network as a way to reduce the overall energy consumption of the network. However, this approach only works under the constraints that the number of CHs still fulfills the requirement that every single node can still relay their packets through one of the selected CHs. The algorithm is based on a spiral sequence generated for arbitrarily deployed nodes. To further minimize the number of CHs, the EEUC proposes the use of some CHs as relay nodes for others under the condition that some CHs run low on their residual energy levels. However, the drawback of the EEUC is that it consumes significant amounts of energy, incurred by cluster formation overhead and fixed-level clustering.
- Power-Efficient and Adaptive Clustering Hierarchy (PEACH): PEACH is proposed by [37] to solve the issue of energy consumed by cluster formation overheads. PEACH uses overhearing (sniffing) characteristics of the wireless communication channel to form clusters without additional overhead. It also supports adaptive multi-level clustering. Besides, PEACH can be used for both location-unaware and location-aware NB-IoT networks. PEACH has been shown to outperform other hierarchical routing protocols such as LEACH in terms of extending the network lifetime [38].
- Hybrid Energy-Efficient Distributed Clustering (HEED): The HEED (Hybrid Energy-Efficient Distributed clustering) protocol periodically selects CHs based on a hybrid parameter made of the node remaining energy as primary parameter and a secondary parameter, such as minimum average distance to a higher number of neighbor nodes. The HEED demonstrates a fairly uniform CH distribution across the network and a low computational complexity O(1), but it suffers from an energy-expensive CH selection process [39]. The HEED also has high inter-cluster traffic which results in energy consumption imbalance susceptible to cause CH nodes to die faster, therefore shortening the lifetime of the network.
- Distributed Weight-based Energy-Efficient Hierarchical Clustering (DWEHC): The authors [40] propose an improved version of the HEED approach called the DWEHC. The DWEHC assigns weight to the primary and secondary CHs selection parameters. The DWEHC approach starts with each node locating its closest neighbors. Based on the received information and its energy parameters, the node works out a hybrid parameter weight value which then serves as decisive in selecting the Cluster Heads. Once more, and like the other approaches, the DWEHC uses a Time Division Multiple Access (TDMA) approach within each cluster as a mechanism for each node within the cluster to send its packet to the CH. However, as the number of nodes increases, the probability of inter-nodes interference increases, network latency deteriorates and so does the network reliability. This is why to minimize the impact of having clusters with congested communication traffic as the NB-IoT network scales, some other approaches such as the Cluster Head Election using Fuzzy logic (CHEF) [41] are proposed.
- Cluster Head Election using Fuzzy logic (CHEF): CHEF [41] proposes the use of a fuzzy logic approach as a mechanism to minimize the energy consumption of CH selection. It also reduces the inter-cluster communication energetic burden on the selected CH by rotating the CH role among nodes for more energy-balanced network performance. The energy level in each node, the concentration (number of nodes present in the vicinity), and the centrality (a value which classifies the nodes based on how central the node is to the cluster) are used as variables in the fuzzy logic rules formulation. However, the model suffers from the following drawbacks,
  1. The fact that the BS has to collect information from all the NB-IoT nodes, results in complex and multiple overheads. This results in additional energy consumption which although it is negligible for a few nodes, becomes significant as the number of nodes increases.
  2. The approach does not cater to the energy consumption caused by the distance measurement between NB-IoT nodes. This is because the CH selection parameter does not feature any signal strength consideration.

### 2.4. Energy-Efficient Network Coding Techniques for NB-IoT Systems

Several approaches have been previously considered to achieve energy efficiency within an NB-IoT system at various network layers [42]. One such technique used at the network layer includes minimizing data traffic using data dissemination, data aggregation (network coding) but also network programming [43]. In a multihop data routing technique, other previous studies on existing network coding techniques found in literature can be broadly classified into two main categories namely the local and the global coding techniques depending on whether the decoding of the aggregated packet is performed at the intermediate NB-IoT nodes or only at the end destination BS. Furthermore, network coding techniques can be classified into intersession and intrasession types depending on the fact that the relay node (CH) in our case only encodes packets from different or the same cluster member nodes respectively [44]. While the intrasession network coding approaches are mainly aimed at reducing packets loss, the intersession network coding approaches are mainly focused on reducing the number of packets re-transmissions as a mechanism to reduce the overall network energy consumption [45]. It is also important to mention that several network coding techniques as found in the literature are also classified in binary(XOR) or random linear(RL) types [44]. Some of the selected few NB-IoT network coding techniques include

- SenseCode: This is a local network coding approach as proposed by [46] that focuses on achieving reliable network performance. SenseCode has demonstrated to significantly reduce the end-to-end packet error rate and is most suitable for highly dynamic environments. However, SenseCode suffers from a high number of network resources among which energy. The performance evaluation of the SenseCode approach under normal static conditions has proven to reduce the packet error rate by 90% as compared to the routing of packets with no network coding. This performance drops to about 75% under highly dynamic environments as evaluated on TinyOS modules using the TOSSIM simulations.
- CodeDrip: This is a technique proposed by [47] to achieve energy efficiency and reliability by allowing the recovery of lost packets using a combination of transmitted packets and decoding on the receiving end. CodeDrip is an improved version of the Drip data dissemination method [48]. The main change from Drip consists of the inclusion of control fields which are necessary for a faster decoding process. However, CodeDrip struggles to cope with high volumes of data packets often encountered in real-time NB-IoT applications.

Given the different identified issues as identified for the various existing approaches as described in the present section, this paper proposes a mixed approach strategy that first tackles the inter-cluster communication scalability issues through the use of an adaptive frequency hopping spread spectrum capable to mitigate interlink interference. Secondly, the proposed approach also proposes an energy-signal-strength-aware CHs selection method with the intent to reduce and balance the overall energy efficiency of the network while offloading the BS. The detailed explanation of this proposed mixed approach is detailed in the next section.

## 3. The Proposed Intelligent Mixed Approach

### 3.1. Hypothesis of the Study

The study was based on the hypothesis that the proposed intelligent mixed approach is capable of switching a common carrier signal among many frequency channels, using a lightweight and secure pseudo-random sequence of a finite period *T* which varies depending on the number of NB-IoT nodes deployed in the cell. The random sequence is made known to other NB-IoT nodes as well as to the Base Station (BS) through an initial broadcast of interest packets. Assuming that only static NB-IoT nodes are considered, this broadcast of interest messages is performed during the initial network deployment. Because of the adaptive nature of the proposed cluster head selection algorithm and the need, therefore, to swap CHs, the broadcasting of interest messages also happens periodically intending to reconfigure the network by selecting new CHs based on a residual energy and signal strength mixed gradient.

The random sequence at a node must be known by neighboring nodes because of the multi-hop cooperative clustering aspect of the proposed method which dictates that some NB-IoT devices within the same cluster are selected to play a relay role to offload the BS. This is expected to reduce the transmission traffic, enhance the Up-link throughput and consequently the up-link data rate, mitigating the inter-cell interference, reducing the number of re-transmission attempts, therefore, enhancing network reliability and energy efficiency.

It is also very important that the pseudo-random sequence generator algorithm is lightweight because of the limited computational, memory but also energy resources of the NB-IoT nodes [49]. Typically, this random number generator algorithm should be implementable on a basic 8-bit microcontroller and its hardware complexity in terms of its gate equivalence (GE) should prove its low complexity. This is a way to ensure that the algorithm is implementable on a small chip area as a single GE in this context is defined to be the silicon area occupied by a single NAND gate.

In summary, this study was based on the hypothesis that the adaptive frequency hopping spread spectrum technique using a lightweight pseudo-random sequence generator, coupled with a cooperative adaptive clustering approach can result in an efficient way to offload the Base Station (BS), and therefore, allows for more NB-IoT nodes to be handled within a single cell. With an efficient way of grouping NB-IoT nodes within a cell and an appropriate cluster head selection based on a composite residual-energy aware and signal strength gradient, in smaller, there is a possibility for better handling of noise and interference mitigation within the cell. This is the hypothesis that this study verifies to primarily enhance network scalability of the NB-IoT network without degrading energy-efficiency performance.

### 3.2. The Mixed Frequency Hopping Spread Spectrum and Smart Clustering Algorithm

#### 3.2.1. The Proposed Frequency Hopping Spread Spectrum Approach

As the first *j* NB-IoT nodes join the network, they send interest messages to the BS. The BS records the Received Signal Strength Indicators (RSSI) of all the *j* nodes and sorts them in ascending order. The lowest RSSI value which represents the NB-IoT node with the lowest level of access to the BS while the NB-IoT node with the highest RSSI represents the node with the highest access to the BS. The RSSIs of the *j* first deployed nodes in their order of arrival form the initial seed of a lagged Fibonacci generator (LFG) series that is used to generate the pseudo-random number sequence. The generated sequence is then used for frequency channel hopping access.

Given a number of *T* of all nodes within the network, the number of elements of the initial seed *j* is selected as per the following considerations:

- A random number generator’s period is the number of new random values that it generates before returning to the start of the sequence.
- The period of the pseudo-random number generated is equal to the number of NB-IoT nodes *T*.
- Maximizing the period ensures that the random numbers generated for each node are from a different period of the LFG.
- The value *j* is selected to provide an optimal balance between the random number generation period and the amount of memory used. This is because the higher the period *T*, the higher the consumption of the already constrained memory space of the NB-IoT nodes.

The proposed algorithm uses a multiplicative LFG (MLFG). For an NB-IoT node with m–bits word-length processor, an MLFG is defined as follows

(1) $$MLFG[j,k,N;\left\{x_{0},x_{1},\ldots ,x_{j-1}\right\}],$$

where

- $N=2^{m}$,
- *j* and *k* are the lag offsets of the past samples in such a way that j>k>0,
- $\left\{x_{0},x_{1},\ldots ,x_{j-1}\right\}$ is the initial seed, and
- the trinomial $x^{j}+x^{k}+1$ which is irreducible and primitive over the Galois Field GF(2).

For a number of NB-IoT nodes *T*, an initial seed size of *j* is chosen such as $T=\left(2^{j}-1\right)\times 2^{m-3}$. This will ensure that the maximum period of the MLFG is equal to the number of NB-IoT nodes *T*. The MLFG pseudo-random values $x_{n}$ are computed by the different nodes as follows

(2) $$x_{n}=\left(x_{n-k}\times x_{n-j}\right)\;Mod\;N.$$

The *j* seed elements on the other hand are selected by the BS upon reception of the interest messages from all the NB-IoT nodes during the initial deployment based on the computed and sorted RSSI values of the received interest messages.

The received RSSI values are computed as follows,

- Through the analysis of the RSSI, it is found that the RSSI value decreases as the distance from the NB-IoT node to the BS increases. Based on this consideration, the free space model (FSM) is used as the RSSI-Based Ranging Model. The FSM is expressed as follows,
  (3) PL=20×log(d)+20×log(f)−27.55,
  where PL, *d* and *f* represent the path loss of the signal energy, the distance between the NB-IoT node and the BS and the transmission frequency being used respectively.
- Furthermore, it is also found that the path loss decreases as the antenna height increases. Based on this consideration, a two-way ground model is proposed as follows,
  (4) $$PL=40\times log\left(d\right)-20\times log\left(h_{n}\right)-20\times log\left(h_{BS}\right),$$
  where $h_{n}$ is the transmitting NB-IoT device height and $h_{BS}$ is the effective height of the Base Station.
- In this study, a two-way ground level model is considered with an average LTE BS effective height of 27.81 m [50] and an average NB-IoT elevation level $h_{n}$ of 7.8 m for most NB-IoT urban applications scenarios [51].
- Considering the NB-IoT antenna gain $g_{a}$ of 6 dBi [52], and a transmit power $P_{TX}$, the RSSI value can be computed as
  (5) $$\left\{\begin{array}{l} RSSI_{dBm}=P_{TX(dB)}+g_{a(dB)}-PL_{dB}\\ RSSI_{dBm}=P_{TX(dB)}+6-PL_{dB} \end{array}\right.$$

The obtained RSSI values are then sorted as follows,

(6) $$x_{n,(0\leq n<j)}=RSSI_{n,(0\leq n<j)}.$$

The initial seed is distributed as part of the downlink broadcast packet to all the NB-IoT nodes. The initial seed is used to form the MLFG sequence that is used by the selected CHs for dynamic frequency hoping and channel selection within the available 200 kHz narrowband bandwidth either deployed in standalone, in-band or guard-band mode. This dynamic frequency hoping and channel selection is performed by CHs as they relay aggregated data packets received from the respective member nodes (MNs) within their clusters to the BS. The proposed dynamic frequency hopping spread spectrum approach is used to mitigate the interlink interference within the cell as the various nodes attempt to reach the BS. It is further supplemented by the clustering approach as described and boosts the overall network security, energy efficiency and robustness of the network but most important, enhances the possibility for more nodes to join the network without compromising its overall network performance.

#### 3.2.2. The Proposed Energy Efficient and Scalable Clustering and Network Coding Approach

**Figure 2:**
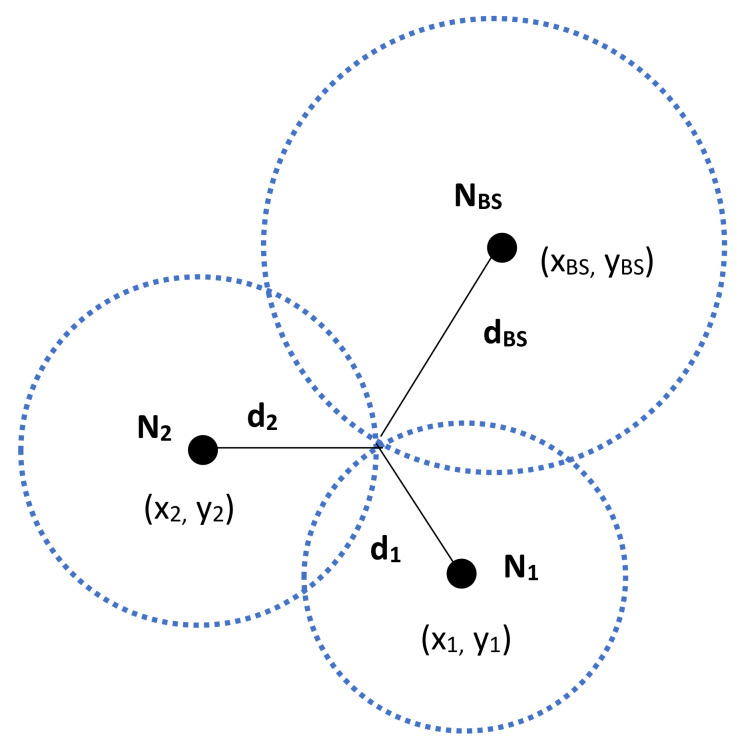
Network Coding for Enhanced Narrowband IoT (NB-IoT) nodes localization approach.

This section describes the proposed cluster groups formation as well as the energy and RSSI aware CHs selection process. The BS in cooperation with two NB-IoT nodes with reliable source powers are equipped with GPS devices and are used as anchor nodes for localization of the other NB-IoT nodes within the cell as follows. The coordinates of the two selected anchor nodes and the BS are known and modelled as $\left(x_{1},y_{1}\right)$, $\left(x_{2},y_{2}\right)$ and $\left(x_{BS},y_{BS}\right)$ respectively. From the broadcast of interest packets as the various nodes join the network, the two nodes and the BS will work out three Euclidean distance values to each of the (j−2) nodes, as per Figure 2, as follows

(7) $$\left\{\begin{array}{l} {\left(x_{n}-x_{1}\right)}^{2}+{\left(y_{n}-y_{1}\right)}^{2}={d_{1}}^{2}\\ {\left(x_{n}-x_{2}\right)}^{2}+{\left(y_{n}-y_{2}\right)}^{2}={d_{2}}^{2}\\ {\left(x_{n}-x_{BS}\right)}^{2}+{\left(y_{n}-y_{BS}\right)}^{2}={d_{BS}}^{2} \end{array}\right.$$

The solution of Equation (7) leads to solving the matrix equation: $AX_{n}=B$ such as

(8) $$\left\{\begin{array}{l} A=\left[\begin{array}{cc} -2(x_{1}-x_{BS}) & -2(y_{1}-y_{BS})\\ -2(x_{2}-x_{BS} & -2(y_{2}-y_{BS}) \end{array}\right]\\ X_{n}=\left[\begin{array}{c} x_{n}\\ y_{n} \end{array}\right]\\ B=\left[\begin{array}{c} {d_{1}}^{2}-{d_{BS}}^{2}-{x_{1}}^{2}+{x_{BS}}^{2}-{y_{1}}^{2}+{y_{BS}}^{2}\\ {d_{2}}^{2}-{d_{BS}}^{2}-{x_{2}}^{2}+{x_{BS}}^{2}-{y_{2}}^{2}+{y_{BS}}^{2} \end{array}\right] \end{array}\right.$$

**Figure 3:**
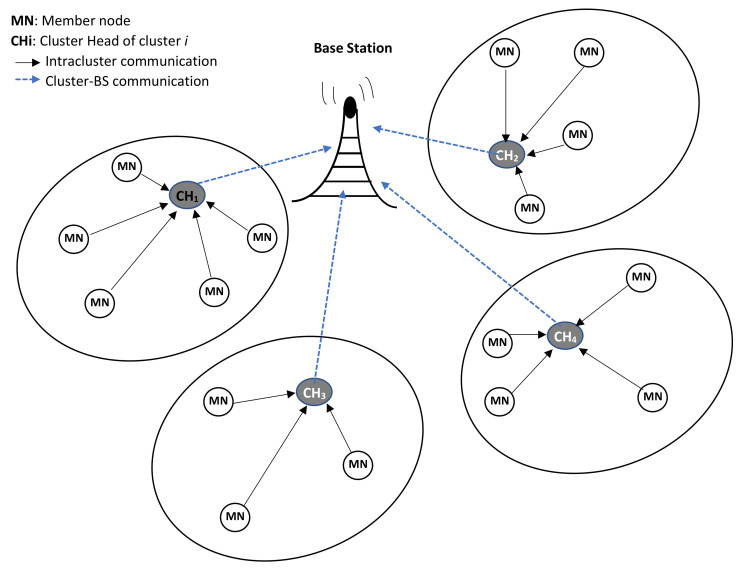
NB-IoT cell cluster formation.

After the geographical localization of nodes within the cell, the BS through its downlink reply packet does not only distribute the initial adaptive spread spectrum frequency hopping seed, it also sends information that geographically groups NB-IoT nodes that are close to each other into clusters. A typical illustration of a typical cluster formation within an NB-IoT cell is shown in Figure 3.

The following steps are taken for clusters formation and CH selection.

- Step 1: Determining the neighbor nodes of each node within the cell by the use of predefined transmission range threshold $d_{th}$ or an optimum number of clusters $p_{opt}$. The problem of least energy number of clusters is a well-studied optimization problem [53] in energy-efficient wireless sensor networks clustering research which is normally formulated as follows,
  (9) $$p_{opt}=\underset{1\leq p\leq j}{arg min}E_{avg}\left(p\right)$$
  subject to: $0\leq P_{t}\leq P_{t_{max}}$. where $E_{avg}\left(p\right)$ is the average energy consumption of the *j* nodes within the cell as modelled in [54] and $P_{t},P_{t_{max}}$ are the transmission power and the maximum transmission powers respectively.
  - It is observed that in an adaptive clusters formation and CHs selection, the process should be dynamic to ensure not only energy efficiency but also energy consumption balance. Because, as the overall average remaining energy efficiency reduces, the number of clusters should be reduced. This means much bigger clusters should be formed to ensure more energy balance and make sure some CHs do not die faster than others. It is also true that the bigger the transmission range threshold used for clusters formation, the smaller the number clusters. This means that the average residual energy is inversely proportional to the transmission range threshold $d_{th}$.
  - On the other hand, the higher the average RSSI value, the more eligible CHs as the more nodes can directly reach the BS. This also translates to a smaller number of clusters which means the higher the transmission range threshold. This means that the average RSSI value is directly proportional to the maximum transmission range threshold.
  The above considerations, lead to a maximum transmission range $d_{th}$
  (10) $$d_{th}=\frac{K\times (MAX\left(d_{n}\right)+MIN\left(d_{n}\right))}{2},$$
  where $K=\frac{RSSI_{avg}}{RP_{avg}}$, $RSSI_{avg}$ is the average RSSI value, and $RP_{avg}$ is the average residual power as observed by the BS after the broadcasting of interest messages during network deployment.
  Our proposed approach opts for the use of the threshold transmission distance to form the different network clusters. Considering the set of all nodes denoted as *N*, the set of neighbour nodes of node *n* denoted as $N_{b}$ can be computed as follows
  (11) $$N_{b}=\sum_{b\in N,b\neq n}d\left(b,n\right)<d_{max},$$
  where d(b,n) is the Euclidean distance between NB-IoT nodes *b* and *n*. This approach comes with the advantage that it avoids the chaining issue.
- Step 2: For every node *n*, all the distances with the neighbors in its cluster is computed as follows
  (12) $$d_{sum}=\sum_{b\in N_{b}}d(n,b).$$
- Step 3: The average residual energy of each cluster *i* denoted as $RE_{CH_{i}}$ is computed as follows,
  (13) $$RE_{CH_{i}}=\frac{1}{sizeof(N_{b})}\times \sum_{b\in N_{b}}RE_{ni}$$
  where $RE_{ni}$ represents the residual energy of node *n* belonging to cluster *i*.
- Step 4: For each formed cluster *i*, the BS computes as a mixed residual energy and signal strength aware cluster head selection gradient $SG_{ni}$ as follows,
  (14) $$SG_{ni}=\alpha \times \left(\frac{RE_{ni}}{RI_{ni}}\right)+\beta \times \left(RSSI_{ni}\right),$$
  where $RI_{ni}$ is the initial energy level of node *n* in cluster *i* and $RSSI_{ni}$ is the RSSI value of node *n* in cluster *i* with respect to the BS. $RSSI_{ni}$ is computed as per Equation (5). On the other hand, the parameters α and β are the cost weights of the residual energy consideration and the reliability of communication aspect respectively. The cost weights α and β are selected in each cluster *i* based on the following criteria,
  (15) $$\left\{\begin{array}{l} \alpha +\beta =1\\ \frac{RE_{CHi}}{RSSI_{CHi}}=\frac{\alpha }{\beta } \end{array}\right.$$
  The criteria in Equation (15) express the fact that, the sum of cost weights (α and β) is equal to unity and the higher the average residual energy in a cluster *i* ($RE_{CHi}$) as compared to the average RSSI of nodes in cluster *i* ($RSSI_{CHi}$), the higher the weight cost α of the residual energy consideration in the definition of the CH selection gradient as compared to the weight cost of the RSSI consideration β and vice-versa.
- Step 5: In each cluster *i*, the node with the highest selection gradient $S_{Gn}$ within a cluster, is selected to be the CH.
- Step 6: Each time a CH receives data packets form various member nodes in its cluster, it applies Random Linear Network Coding (RLNC) (data aggregation) before transmitting it to the BS. In the typical two-hops clustering approach as considered by this study, the focus of this study is mainly on the global network coding approaches as the data aggregation point is the CH after which the aggregated data is immediately forwarded to the BS where its decoding occurs. Furthermore, the proposed network coding approach is an intersession network coding scheme in which data packets form various member nodes within the network are aggregated together before being transmitted as a single packet to the BS. The *m* independent data packets ($d_{i}$) from the *m* member nodes of the network cluster *i* are linearly combined by means of *m* random coefficients ($C_{i}$) from the GF of order $2^{S}$. The value *S* is a positive number equal to the size of a single NB-IoT packet. The *m* packets are combined to form a single aggregated packet of the same size *S* as each one of them. A GF as generated by each CH is also made know to the BS for the sake of the decoding process. The aggregated (encoded) packet *A* is obtained as follows,
  (16) $$A=\sum_{i=1}^{m}C_{i}\times d_{i}.$$
  The key challenge associated with the network coding technique is that the decoding of the encoded packet is performed by Gaussian elimination and, therefore, requires the reception of at least *m* packets to occur. The decoding process on the BS side is performed by forming a m×m coefficient matrix $C_{m\times m}$ from the received *m* encoded packets and by reducing it using the row-echelon form. Considering that the linear independence of encoded packets, the set of *m* linearly independent equations is solved to retrieve each originally encoded packet. One key advantage of this proposed RLNC approach is that the choice of encoding coefficients form a GF, enhances the probability of linear independence among the encoded packets which then translates in high decoding reliability.

The combination of the proposed dynamic frequency hoping and the energy-RSSI aware clustering with network coding approaches form the mixed proposed strategy for enhancing the overall network scalability, energy efficiency and latency of the network. A R2019b MATLAB version has been used to implement and evaluate the performance of the proposed NB-IoT Mixed Approach with Network Coding (N-MANC) in comparison the NB-IoT with the adaptive frequency hopping spread spectrum at PHY layer only (N-AFHSS) [55], then with respect to the NB-IoT with LEACH clustering at the network layer (N-LEACH) [56], and finally with the NB-IoT Mixed approach with no network coding (N-MANNC). The main performance comparison metrics considered are network scalability, energy efficiency, network reliability and network lifetime; as described in Section 4.

## 4. Performance Evaluation and Obtained Results

This section presents the performance evaluation process in terms of the simulation parameter considerations, the simulation scenarios as well as the obtained performance results and their discussion.

### 4.1. Evaluation Set-Up

The performance evaluation of the proposed N-MANC approach versus the N-AFHSS, the N-LEACH and the N-MANNC is performed under the network considerations as summarized in Table 1. It is important to note that a simulation is conducted for each of the compared approaches with the objective to elucidate the actual contribution of a specific addition or modification to an approach in terms of network scalability, energy efficiency, network reliability and network lifetime. The proposed approach as well as the approaches to which it is compared are all simulated under the same simulation conditions to ensure fair performance comparison.

It is also important to note that eNodeBs of the LTE network are often designed to handle QPSK, 16 QAM and 64 QAM modulations schemes for the Downlink (DL) communications. However, due to the limited computational ability of the NB-IoT node, often micro-controller based in the majority of IoT applications such as smart water, electricity metering etc.), the modulation scheme of choice is the QPSK as specified in Table 1 [58].

### 4.2. Real-Life Application Scenario under Simulation

The realistic case scenario considered in our study is one of the smart water metering applications. As we know, the water meter, unlike the other utility meters, is very energy-critical because of the regulation in many countries like South Africa which forbids to bring electrical lines on a water utility. Most water supply companies request a guaranteed minimum of 10 years lifetime of smart water meter design. The smart water meters are fixed sensor nodes and the ones considered for this research are assumed to have a Tunnel Magneto Resistance (TMR) active magnetic sensor to detect and convert the turning of the mechanical register. Every turning cycle of the mechanical register corresponds to a well-defined water flow value and results in a digital pulse transition on the output of the TMR sensor. The digital processor of the smart water meter counts the number of digital pulses it receives. The water volume flow is computed based on the total number of counted TMR digital pulses. The smart water meter is also able to detect any tampering activities. There is an NB-IoT radio module in each smart water meter and the assumption that there is one smart water meter per household. The choice of 1000 to 150,000 NB-IoT nodes represents the number of smart water meters and other battery-powered environment monitoring smart devices such as geyser leak detectors, door sensors, room occupancy detection sensors, fire detection sensors, water tank level monitoring systems, smart fridges, smart TVs etc. The set of nodes considered in this study are normally static sensors and limited in terms of energy resources. With the increasing number of IoT devices applications, this study caters for up to 150,000 nodes within the geographical area of an NB-IoT cell. In terms of the data traffic, data packets are sent on an average of 1 packet per day by these devices. Hence our consideration of up to 3960 rounds of transmission packets for 11 years of operation. The daily data packet sent as an uplink packet by each smart water meter contains information about the device’s status (water meter daily consumption, any sensed possible leaks as well as the remaining battery level percentage of the smart water meter for example). The other environment monitoring smart devices are also assumed to send their daily status once a day either in the case of a triggering event (alarm) or around midnight for a summary daily report. The traffic data model used in our simulation is therefore made up of a combination of deterministic daily packet transmissions coupled with a uniformly distributed stochastic event-triggered packet transmission. In essence, every single node has a minimum of 1 packet per day.

### 4.3. Obtained Results, Analysis and Discussion

This subsection presents the obtained performance evaluation results for the various considered simulation scenarios.

#### 4.3.1. Energy Consumption Performance Results

**Figure 4:**
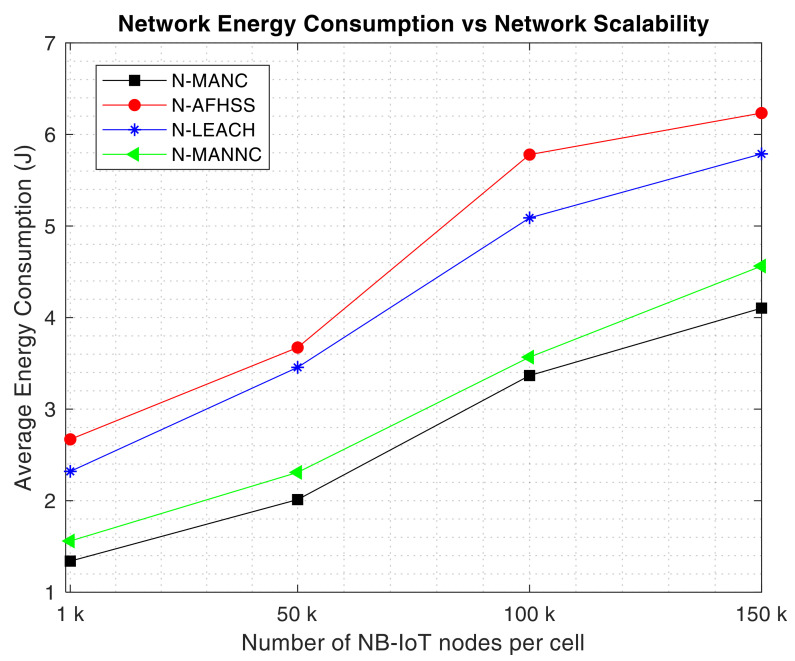
Average energy consumption versus network scalability.

First, the average energy consumption performance of the proposed N-MANC mixed approach as the network scalability is progressively increased, is assessed with respect to the N-AFHSS, N-LEACH and N-MANNC. The obtained results for a single communication period (*P*) are captured in Figure 4.

It is important to mention that the results shown in Figure 4, have been obtained by simulating each of the approaches under comparison, one at a time and for a transmission period *T* of one hour, with a varying cell density from 1000 to 150,000 nodes, and each node having an initial energy level of 7 J. As it can be observed in Figure 4, the average energy consumption within the cell is increasing for all the four approaches, as more and more nodes are added within the cell. This behavior is expected because as more and more devices join the network, the probability of successful up-link transmission reduces because of more and more interlink interference. However, as it can be noticed, despite the increase in the average energy consumption of the network, the proposed N-MANC maintains a way lower energy consumption as compared to the N-AFHSS and the N-LEACH and maintains quite a significantly lower energy consumption as compared to the N-MANNC. The N-MANC consumes an average of 41.04% less energy than the N-AFHSS, 35.01% less than the N-LEACH, and only 9.81% less than the N-MANNC. This is justified by the fact that thanks to its mixed approach strategy tapping in both network reliability of the AFHSS, the network clustering network density reduction of the clustering and the number of transmitted packets reduction as added by the network coding approach as proposed; the N-MANC saves a significant amount of energy as compared to the N-AFHSS which only enhances the network reliability and the N-LEACH which only addresses the network density reduction aspect. It is important to notice the 9.81% energy consumption reduction as contributed by the random linear network coding approach as compared to the N-MANNC which does not have any network coding. This sufficiently demonstrated that packet aggregation with enhanced reliable communication as a pre-requisite can help save a great deal of energy within the NB-IoT network, despite the increasing number of nodes within the cell. This demonstrates that the proposed N-MANC scales in a more energy-efficient manner as compared to the N-AFHSS, the N-LEACH, and the N-MANNC.

#### 4.3.2. Network Reliability Performance Evaluation Results

**Figure 5:**
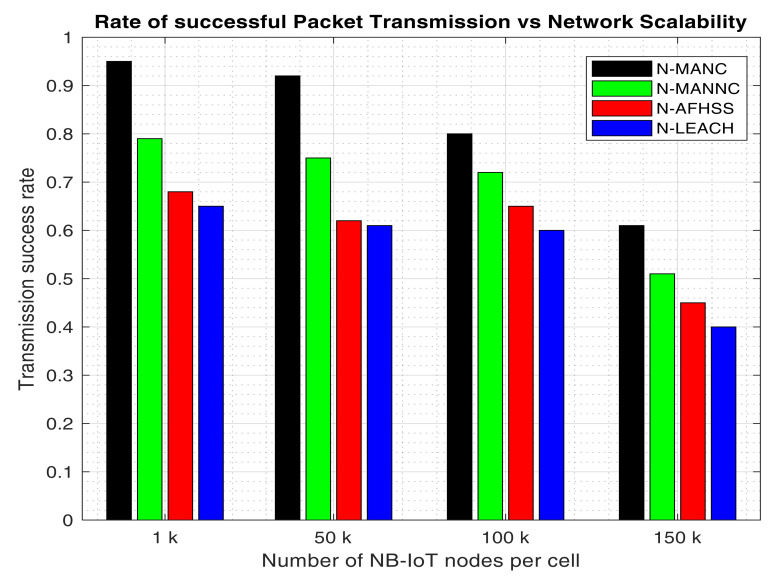
Packets transmission success rate versus network scalability.

The average rate of successful up-link transmissions is assessed for comparison of the proposed N-MANC to the other approaches (N-AFHSS, N-LEACH, and the N-MANNC). This average rate of successful transmissions is computed as the various number of nodes per cell is increased to assess the impact of the network scalability on the network transmission reliability of the network. The obtained results are illustrated in Figure 5.

It can be observed from the bar graph in Figure 5 that the rate of successful transmissions drops as the number of NB-IoT nodes increases in the cell or all the assessed approaches. However, the proposed mixed approach (N-MANC) maintains the highest probability of successful transmission as compared to the N-MANNC, the N-AFHSS, and the N-LEACH respectively. On average the proposed N-MANC maintains a 5.86% higher transmission success rate as compared to the N-MANNC, 10.83% higher than the N-AFHSS, and 55.84% higher than the N-LEACH respectively. It is important to notice that the N-AFHSS approach outperforms the N-LEACH in terms of uplink transmission reliability. This is because the N-AFHSS unlike the N-LEACH is primarily designed to mitigate the inter-link interference which is one of the main causes of unsuccessful up-link transmissions; unlike the N-LEACH which is mainly focused on reducing the network communication traffic to primarily enhance its energy efficiency. It is however important to note the N-LEACH approach also contributes towards improving the network communication reliability and, therefore, the proposed mixed N-MANC demonstrates way better transmission success rate as its taps into the benefits of the N-AFHSS as well as the N-LEACH approach. On the other hand, the improved transmission reliability enhances the RLNC performance in terms of its decoding process. This results in the RLNC further contributing towards enhanced network transmission reliability as compared to the mixed approach with no network coding (N-MANNC) despite the increase in the number of nodes within the cell.

#### 4.3.3. Network Lifetime Performance Evaluation Results

**Figure 6:**
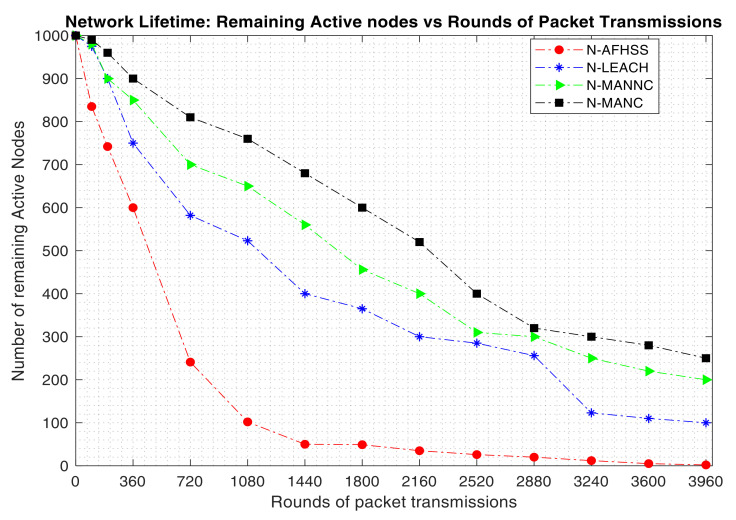
Network lifetime: remaining nodes versus round of transmission packets.

It was also crucial to assess the contribution of the proposed approach towards enhancing the network’s lifetime. To assess the network lifetime performance, the number of simulation runs have been performed to compute the number of remaining active nodes as the rounds of packet transmissions is increased to evaluate at what rate the network dies when the proposed N-MANC approach is used as compared to the N-LEACH, N-MANNC and the N-AFHSS. The obtained results are shown in Figure 6.

It can be observed in Figure 6 that the number of remaining active nodes reduces as the number of rounds of packet transmissions increases. Since most of the NB-IoT applications work based on periodic packet transmissions, the rounds of packet transmissions often correspond to the number of packet transmission periods. For a typical smart water metering application with a daily water consumption packet transmission, the maximum round of packet transmissions considered for our simulations (3960) corresponds to a total of almost 11 years of operation for a yearly number of days of roughly 365 days; considering that the NB-IoT node is in deep sleep mode for the rest of the day where it consumes almost no power.

It can also be noticed in Figure 6 that the number of remaining active NB-IoT nodes when the proposed N-MANC approach is used remains always higher as compared to when the N-MANNC, the N-LEACH, and the N-AFHSS are used respectively. it is also important to mention that with the use of the N-AFHSS, the network dies much faster as compared to the others (N-LEACH, N-MANNC, and N-MANC) respectively. This demonstrates that the energy consumption reduction of the proposed mixed approach (N-MANC) as depicted in Figure 4 coupled with its enhanced network reliability as shown in Figure 5; considerably contributes towards significantly extending the NB-IoT network lifetime.

**Figure 7:**
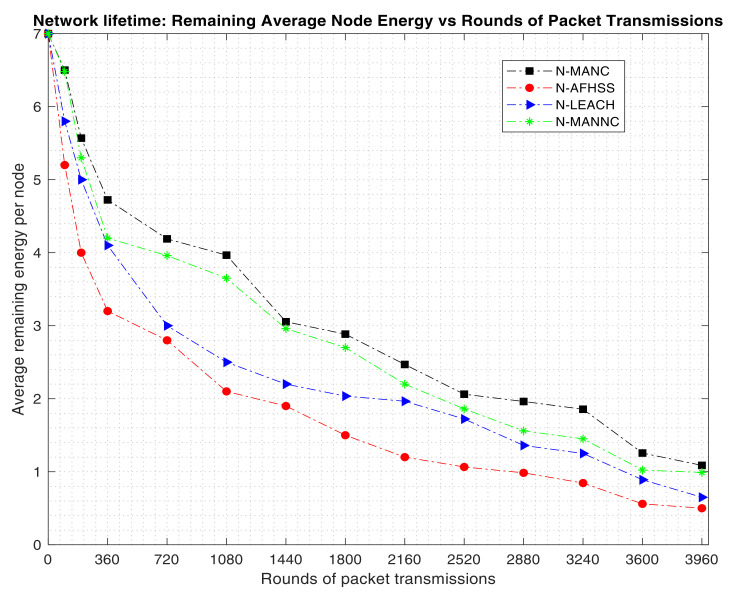
Network lifetime: remaining average energy versus round of transmission packets.

The results in Figure 6 are further confirmed by the results of the evaluation of the average remaining energy per node of the proposed N-MANC approach as compared to the N-MANNC, N-LEACH, and the N-AFHSS; as shown in Figure 7.

The results in Figure 7 clearly show that the same way the number of remaining active nodes in Figure 6 as more rounds of packets are transmitted, the same way the average remaining energy per node also reduces. The results in Figure 7 also show that the N-MANC approach maintains the highest average remaining energy per node for all the considered rounds of packet transmissions as compared to the N-MANNC, the N-LEACH, and the N-AFHSS respectively.

## 5. Conclusions

A novel NB-IoT Mixed spread spectrum and clustering Approach with Network Coding (N-MANC) is presented in this research article to enhance the NB-IoT network scalability while improving its energy efficiency, network reliability, and network lifetime performance. The N-MANC is proposed as a step towards enabling the massive connections of IoT objects to the cellular network (5G and beyond) within the licensed band. After establishing through literature survey that this enhanced scalability of the NB-IoT network is a cross-layers effort, the present research work has proposed a mixed approach which at the Physical layer (PHY) proposes an adaptive frequency hopping spread spectrum technique using a lightweight pseudo-random sequence generator coupled with the clustering and network coding techniques at the network layer. The proposed approach is described in detail in terms of its implementation and underlying concepts.

After evaluation of the proposed approach, the obtained and discussed results lad to the conclusion that the proposed N-MANC approach helps mitigates the scarcity of the available spectrum coupled with the interlink interference to enable more NB-IoT nodes to join the network cell while ensuring an offloading of the BS using an energy and RSSI aware formation of network clusters and selection of CHs. It can be further concluded that the offloading of the BS together with the aggregation of data packets at each CH using the proposed RLNC technique, has demonstrated the ability to significantly improve the energy efficiency, network reliability and network lifetime of the network as compared to the N-AFHSS (with adaptive frequency hopping spread spectrum only), the N-LEACH (with energy-efficient clustering only), and N-MANNC (Mixed approach with no network coding) respectively.

## 6. Future Work

The proposed NB-IoT Mixed spread spectrum frequency hopping and clustering approach with network coding (N-MANC) has demonstrated enhancement of the network scalability while improving the overall energy efficiency, transmission reliability and lifetime of the NB-IoT. Therefore, our future research work will consist of firstly implementing and assessing the proposed N-MANC on-chip for the same considered application scenario of smart water metering coupled with other application scenarios such as environmental monitoring sensor network. This implementation will help demonstrate and validate the proposed approach.

## Figures and Tables

**Table 1:** Key simulation parameters.

Transmitting device $\left(h_{n}\right)$	7.8 m
average height	
Effective Height	27.81 m
of the BS $\left(h_{BS}\right)$	
Antenna Gain $\left(G_{a}\right)$	6 dBi
System bandwidth	200 kHz
Carrier frequency	900 MHz
Residual Frequency Error	±50 Hz
Average NB-IoT cell radius range	10 km (urban)
Number of Nodes per cell (T)	[1 k, 10 k, 50 k, 100 k, 150 k]
Communication period (*P*)	1 h
Maximum payload length	1600 bytes
Localization Technique	RSSI-based
Channel estimation for NPDCCH	Sequential
	Channel Estimation in the
	Presence of Random Phase Noise [57]
Interference Rejection Combiner	MRC
Number of Tx antennas	1
Number of Rx antennas	2
Duplex mode	Half-Duplex Frequency
	division Duplex
	(HD-FDD)
Initial NB-IoT node	3×AA Batteries
energy (Power capacity)	Nominal voltage =
	3×1.5=4.5 V
	Each AA has 1500 mAh
	Initial Energy = 6.75 J
BS power	43 dBm
Receiving Sensitivity level	−90 dBm
Time offset period	2.5μs
Channel Model	Time-invariant slow fading
	with random phase noise
	following Additive White
	Gaussian Noise (AWGN)
	distribution
Slow or	Static nodes
almost no nodes mobility	No fading channel
considered	consideration
LTE Modulation Scheme	Quadrature Phase Shift
	Keying (QPSK)

## Abbreviations

The following key abbreviations are used in this manuscript:

NB-IoT:	Narrowband Internet of Things
NB-IoT node:	Device equipped with a processor
	and an NB-IoT modem such as a sensor node,
	a smart water meter, etc.
N-MANC:	Narrowband Internet of Things
	Mixed spread spectrum and clustering
	Approach with Network Coding
N-AFHSS:	Narrowband Internet of Things
	with Adaptive Frequency Hopping
	and Spread Spectrum Technique
N-LEACH:	Narrowband Internet of Things
	with Low Energy Adaptive Clustering
	Hierarchy protocol
N-MANNC:	Narrowband Internet of Things Mixed spread
	spectrum and clustering Approach
	with No Network Coding
LTE:	Long Term Evolution
3GPP:	Third Generation Partnership Project
RSSI:	Received Signal Strength Indicator
